# Methadone Dosage and Plasma Levels, SNPs of OPRM1 Gene and Age of First Drug Use Were Associated With Outcomes of Methadone Maintenance Treatment

**DOI:** 10.3389/fgene.2018.00450

**Published:** 2018-10-29

**Authors:** Sufang Peng, Haifeng Jiang, Jiang Du, Shuxing Lin, Shujun Pan, Shunying Yu, Min Zhao

**Affiliations:** ^1^The Collaborative Innovation Center for Brain Science, Shanghai Mental Health Center, Shanghai Jiao Tong University School of Medicine, Shanghai, China; ^2^Shanghai Key Laboratory of Psychotic Disorders, Shanghai, China; ^3^Brain Science and Technology Research Center, Shanghai Jiao Tong University, Shanghai, China

**Keywords:** methadone maintenance treatment, OPRM1, ABCB1, methadone dosage, plasma drug concentration

## Abstract

**Objective:** To explore the association between methadone dosage, plasma drug concentration, SNPs of μ-opioid receptor gene (*OPRM1*), ATP-binding cassette subfamily B member 1 gene (*ABCB1*), and methadone maintenance treatment (MMT) response.

**Method:** A total of 240 Chinese Han participants receiving MMT were recruited from Shanghai. Nine single nucleotide polymorphisms (SNPs) of the *OPRM1* gene and three SNPs of the *ABCB1* gene were genotyped, plasma methadone concentration was detected, and a morphine urine test was taken from all subjects.

**Results:** Methadone dosage, plasma methadone concentration, and negative rate of morphine urine test of retention participants were significantly higher, although the addiction severity index (ASI) was not significantly different between the two groups. A allele and AA genotype carriers of rs562859 (*OPRM1* gene) had better compliance of MMT, and AA genotype carriers had a higher negative rate of morphine urine test. However, the difference was not significant after adjusting influence factors (age, sex, and methadone dosage). GG genotype carriers of rs3192723 (*OPRM1* gene) had a significantly lower negative rate of morphine urine test, and the difference was still significant after adjusting influence factors. Logistic regression analysis showed that methadone-free trough concentration (OR = 0.910, *p* = 0.023) and AA genotype of rs526859 (OR = 0.580, *p* = 0.037) were associated with better compliance of MMT. After Bonferroni correction, only free trough concentration of methadone was negatively correlated with MMT compliance. The SNPs rs6912029 (OR = 0.021, *p* = 0.066) and rs6902403 (OR = 0.910, *p* = 0.007) of the *OPRM1* gene, age at first use (OR = 1.118, *p* = 0.005), and average methadone dosage (OR = 1.033, *p* = 0.045) were associated with MMT effect. After Bonferroni correction, average methadone dosage was no longer correlated with MMT effect.

**Conclusion:** Dosage of methadone, plasma methadone concentration, several SNPs (rs3192723, rs6912029, rs6902403) of the *OPRM1* gene, and age of first drug use were associated with better MMT outcomes.

## Introduction

Heroin dependence is an important public health problem, which increases the medical burden, brings about social problems, and increases the cost of social security with the gradually increasing number of users (Yang et al., 2016). Methadone maintenance treatment (MMT) is the most commonly used method to treat heroin dependence. Methadone combines competitively with opioid receptors and balances the level of opioid peptide, which is the basis for analgesic and sedation effects, similar to what is observed with heroin. Methadone causes minor euphoria and side effects and can remit withdrawal symptoms, and subsequently reducing cravings and illegal drug use (Bertschy, 1995; Effective medical treatment of opiate addiction., 1998; Ward et al., 1999; Bament et al., 2004). However, in the clinic, there was a great individual difference in the effect and dosage of methadone (Bell et al., 2006). A previous study showed that 30–80% of the patients experienced unsatisfactory therapeutic effects, if the information of whether or not patients stayed in MMT or whether or not patients used opium illegally was considered as the evaluation index (USGA Office, 1990). The effect of MMT was influenced by individual hereditary factors (such as genetic polymorphism of metabolic enzymes, pharmacokinetics of methadone), clinical features, dosage, and duration of methadone administration (Maxwell and Shinderman, 1999; Cacciola et al., 2001; Farré et al., 2002; Pérez de los Cobos et al., 2007; Elkader et al., 2009; Epstein et al., 2009; Zhang et al., 2011). In the past years, the genetic factors of MMT have attracted more and more attention.

The μ opium receptor (OPRM1), encoded by the *OPRM1* gene, is the main action site of methadone. Previous studies indicated that *OPRM1* polymorphism may be associated with treatment response of methadone, but the results were contradictory. The SNP rs558025 was found to be associated with MMT outcome (Levran et al., 2013). G allele carriers of rs1799971 and C allele carriers of rs2075572 required higher methadone dosage (Wang et al., 2012; Bauer et al., 2015). However, the studies conducted by Crettol et al. and Hung et al. failed to find an association between *OPRM1* polymorphism and MMT effect or methadone dosage (Crettol et al., 2008; Hung et al., 2011).

P-glycoprotein, encoded by the ATP-binding cassette sub-family B member 1 gene (*ABCB1*), is the main transporter of methadone. When the activity of P-glycoprotein increased, more extracellular transfer of methadone occurred, and the intracellular drug concentration decreased. A previous study showed that genetic polymorphism of *ABCB1* was associated with MMT effect and methadone dosage (Levran et al., 2008). The SNPs rs1045642 and rs1128503 were associated with methadone concentration in plasma but were not associated with MMT effect (Crettol et al., 2006; Levran et al., 2013). There were also contradictory results. The studies conducted by Lötsch et al. and Fonseca et al. failed to find an association between *ABCB1* SNPs and MMT effect or methadone concentration in plasma (Lötsch et al., 2006; Fonseca et al., 2011).

The initial methadone dosage of MMT was determined by a clinical doctor, in terms of the period of heroin usage, daily dosage, method of usage, last dosage, and the usage time. Later, the methadone dosage was adjusted based on the actual situation, such as withdrawal symptoms, missed dose, and positive urine test. Generally, the recommended methadone dosage was more than 60 mg/d, and 80–100 mg/d was better (Eap et al., 2002; Lehotay et al., 2005). Maxwell's study found that people who take more than 100 mg/d of methadone experienced better treatment effects (Maxwell and Shinderman, 2002). In China, the recommended methadone dosage was also more than 60 mg/d; however, a significant number of patients in MMT took a lower dosage, and the doses varied widely between individuals. This resulted in withdrawal symptoms and poor treatment effect (Wang et al., 2007; Chen et al., 2010). Xiaobing Cao's study showed that dropout patients, who had shorter maintenance time and unsatisfied treatment effect, took a lower methadone dosage (Cao et al., 2010). Even when the patients took the same dosage, the concentration of methadone in plasma was widely varied between individuals, which resulted in a great difference in the response to the treatment (Li et al., 2008). A higher plasma methadone concentration would result in better control of withdrawal symptoms (Ries et al., 2009).

On the basis of previous studies about the association between gene/plasma drug concentration and MMT effect, we promote the hypothesis that polymorphisms of the *OPRM1* and *ABCB1* genes may influence the plasma methadone concentration and MMT effect. The MMT effect may be associated with the genotype, plasma drug concentration, and methadone dosage. In order to verify the hypothesis mentioned above, we tested nine SNPs of *OPRM1* (rs3192723, rs675026, rs562859, rs6912029, rs2236256, rs2236257, rs2236258, rs2236259, and rs6902403) and three SNPs of *ABCB1* (rs1045642, rs1128503, and rs2032582). We used the completion/incompletion of the 6 months of MMT as the treatment compliance index and the negative rate of morphine urine test as the treatment effect index and analyzed the effect of methadone plasma concentration and *ABCB1*/*OPRM1* gene polymorphisms on MMT outcomes (compliance/effect). The results of this study may guide the clinical work of MMT in China.

## Results

Clinical features of participants are summarized in Table 1. No Hardy–Weinberg equilibrium deviation was found in the genotype distribution of any SNP in the retention or dropout group (*p* > 0.05).

**Table 1:** Clinical features of participants.

	**Retention (*n =* 144)**	**Dropout (*n =* 96)**	**total (*n =* 240)**	***p***
Gender:man, *n* (%)	111 (77.1)	75 (78.1)	186 (77.5)	0.850
Woman, *n* (%)	33 (22.9)	21 (21.9)	54 (22.5)	
Ages, years (SD)	41.0 (8.4)	40.8 (8.6)	40.9 (8.5)	0.920
**MARITAL STATUS**				
Unmarried *n* (%)	55 (38.2)	35 (36.4)	90 (37.5)	0.805
Married/remarried *n* (%)	49 (34.0)	35 (36.4)	84 (35)	
Divorced/widowed *n* (%)	39 (27.1)	26 (27.1)	65 (27)	
**EDUCATION**				
Junior high school/under junior high school, *n* (%)	88 (61.1)	63 (65.6)	151 (62.9)	0.388
High school or above, *n* (%)	56 (38.9)	33 (34.4)	89 (37.1)	
Years of education, years (SD)	9.96 (1.9)	9.79 (1.9)	9.9 (1.9)	0.501
**MANNER OF WORKING**				
Full-time/regular part-time working, *n* (%)	21 (14.6)	25 (26.0)	46 (19.1)	0.182
Unemployed, *n* (%)	103 (71.5)	66 (68.8)	169 (70.4)	
The age of first use heroin, years (SD)	29.8 (8.9)	28.2 (8.1)	29.2 (8.6)	0.165
Heroin‘ use before enrollment, days (SD)	24.8 (9.4)	26.0 (8.3)	25.3 (9.0)	0.292
Heroin use,years (SD)	9.3 (4.3)	9.6 (4.7)	9.4 (4.5)	0.622
Duration of fist heroin use and dependence,month (SD)	3.52 (5.91)	4.15 (7.16)	3.77 (6.43)	0.464
**ABUSE OF**				
Intravenous injection, *n* (%)	97 (67.4)	66 (68.8)	163 (67.9)	0.847
Iron absorption, *n* (%)	22 (15.3)	11 (11.5)	33 (13.8)	
Snorting, *n* (%)	14 (9.7)	12 (12.5)	26 (10.8)	
Years of smoking, (SD)	16.5(9.5)	18.3 (9.2)	17.2 (9.4)	0.155
History of imperative detoxification *n* (%)	83 (57.6)	44 (45.8)	127 (52.9)	0.101
History of detoxification via reeducation, *n* (%)	98 (68.1)	70 (72.9)	188 (70.0)	0.333
History of illegal activity, *n* (%)	39 (27.1)	22 (22.9)	61 (25.4)	0.665

### Addiction severity index, methadone dosage, drug concentration in plasma, and negative rate of morphine urine test

A total of 170 blood samples before and after taking methadone were collected from the participants, 15 samples of which were excluded because of hemolysis and bad anticoagulant. Eventually, 155 samples (105 in retention group and 50 in dropout group) were analyzed for the concentrations of methadone. As shown in Table 2, there was no significant difference in the addiction severity index (ASI; *p* > 0.05) between retention and dropout participants. However, the methadone dosage, drug concentration in plasma, and negative rate of morphine urine test of the retention participants were significantly higher than those of the dropout participants (*p* < 0.05; Table 3).

**Table 2:** Addiction severity index of retention and dropout participants.

	**Retention (*n =* 144)**	**Dropout (*n =* 96)**	**Total (*n =* 240)**	***P***
**ADDICTION SEVERITY INDEX ME(SD)**					
Physical health	0.11 (0.21)	0.12 (0.24)	0.11 (0.22)	0.52
Vocational function	0.86 (0.25)	0.82 (0.28)	0.84 (0.26)	0.22
Use of alcohol	0.05 (0.10)	0.06 (0.12)	0.06 (0.11)	0.40
Use of drugs	0.21 (0.08)	0.23 (0.08)	0.22 (0.08)	0.28
Legal question	0.06 (0.09)	0.07 (0.10)	0.96 (0.10)	0.38
Family function	0.14 (0.14)	0.13 (0.15)	0.14 (0.14)	0.40
Psychological health	0.13 (0.19)	0.11 (0.19)	0.12 (0.19)	0.34

**Table 3:** Methadone dosage, drug concentration in plasma, negative rate of morphine urine test of retention and dropout participants.

**Items, Me (SD)**	**Retention (*n =* 105)**	**Dropout (*n =* 50)**	**Total (*n =* 155)**	***p***	**Cohen's d**
Average dosage of methadone, mg/d	44.22 (23.11)	36.46 (34.09)	41.07 (28.22)	***0.03***	0.27
Free trough concentration, ng/ml	13.68 (8.71)	9.37 (5.27)	12.28 (8.00)	***0.00***	0.60
Total trough concentration, ng/ml	239.16 (142.62)	177.93 (112.28)	219.42 (136.28)	***0.01***	0.48
Free peak concentration,ng/ml	20.83 (12.59)	15.46 (7.94)	19.10 (11.56)	***0.01***	0.51
Total peak concentration, ng/ml	363.43 (212.34)	291.37 (157.40)	340.18 (198.68)	***0.03***	0.39
negative rate of morphine urine test	0.83 (0.21) (*n =* 113)	0.49 (0.39) (*n =* 67)	0.71 (0.33) (*n =* 180)	***0.00***	1.09

### OPRM1/ABCB1 SNP genotypes and compliance of MMT

In this study, a total of 166 participants provided blood samples for DNA extraction (111 in the retention group and 55 in the drop-out group). Further details are provided in the Supplementary Material. As shown in Table 4, there was a difference in the distribution of rs562859 (*OPRM1* gene) between retention and dropout groups. A allele and AA genotype were the protective factors for the compliance of MMT [*p* < 0.05, OR (95%CI) was 0.58 (0.34–0.97)]. However, the difference was no longer significant after adjusting age, sex, and methadone dosage (*F* = 1.30, *p* > 0.05). There was no difference in the distribution of allele or genotype of SNPs of the *ABCB1* gene (*p* > 0.05).

**Table 4:** Alleles and genotypes of the *OPRM1* and *ABCB1* genes.

**SNP**		**Allele**		**χ^2^**	***p***	**ϕ**	**OR [95% CI]**	**genotype**			**χ^2^**	***p***	**ϕ**
***OPRM1***													
		A	G					AA	AG	GG			
rs3192723	Retention *n =* 144	57 (0.26)	165 (0.74)	0.70	0.40	0.05	1.24 [0.75-2.06]	3 (0.03)	51 (0.46)	57 (0.51)	1.95	0.38	0.11
	Dropout *n =* 96	33 (0.30)	77 (0.70)					4 (0.07)	25 (0.46)	26 (0.47)			
		C	T					CC	CT	TT			
rs675026	Retention *n =* 144	200 (0.92)	18 (0.08)	1.36	0.24	0.08	1.81 [0.66-5.04]	93 (0.85)	14(0.13)	2(0.02)	1.44	0.49	0.09
	Dropout *n =* 96	101(0.95)	5 (0.05)					48 (0.91)	5 (0.09)	0 (0.00)			
		A	G					AA	AG	GG			
rs562859	Retention *n =* 144	178 (0.80)	44 (0.20)	4.28	***0.04***	0.13	0.58 [0.34-0.97]	73 (0.66)	32 (0.29)	6(0.05)	6.82	***0.03***	0.20
	Dropout *n =* 96	77 (0.70)	33 (0.30)					25 (0.45)	27 (0.49)	3 (0.06)			
		G	T					GG	GT	TT			
rs6912029	Retention *n =* 144	129 (0.59)	91 (0.41)	0.31	0.58	0.04	1.14 [0.71-1.83]	36 (0.33)	57 (0.52)	17 (0.15)	1.39	0.50	0.09
	Dropout *n =* 96	68(0.618)	42 (0.382)					18 (0.33)	32 (0.58)	5 (0.09)			
		A	C					AA	AC	CC			
rs2236256	Retention *n =* 144	33 (0.15)	189 (0.85)	0.96	0.33	0.06	1.35 [0.74-2.47]	6 (0.05)	21 (0.189)	84 (0.76)	0.83	0.66	0.07
	Dropout *n =* 96	21 (0.19)	89 (0.81)					4 (0.07)	13 (0.24)	38 (0.69)			
		C	G					CC	CG	GG			
rs2236257	Retention *n =* 144	37 (0.17)	187 (0.84)	0.06	0.80	0.02	0.92 [0.49-1.72]	1 (0.01)	35 (0.31)	76 (0.68)	0.50	0.78	0.06
	Dropout *n =* 96	17 (0.16)	93 (0.84)					0 (0.00)	17 (0.31)	38 (0.69)			
		C	T					CC	CT	TT			
rs2236258	Retention *n =* 144	87 (0.39)	135 (0.61)	1.97	0.16	0.09	1.40 [0.88-2.20]	12 (0.11)	63 (0.57)	36 (0.32)	2.48	0.29	0.12
	Dropout *n =* 96	52 (0.47)	58 (0.53)					10 (0.18)	32 (0.58)	13 (0.24)			
		C	T					CC	CT	TT			
rs2236259	Retention *n =* 144	137 (0.62)	83 (0.38)	3.90	0.05	0.13	0.63 [0.40-1.00]	43 (0.39)	51 (0.46)	16 (0.15)	4.97	0.08	0.17
	Dropout *n =* 96	56 (0.51)	54 (0.49)					12 (0.22)	32 (0.58)	11 (0.20)			
		C	T					CC	CT	TT			
rs6902403	Retention *n =* 144	174 (0.79)	46 (0.21)	0.03	0.87	0.01	0.95 [0.54-1.68]	68 (0.62)	38 (0.34)	4 (0.04)	0.41	0.82	0.05
	Dropout *n =* 96	83 (0.78)	23 (0.22)					33 (0.62)	17 (0.32)	3 (0.06)			
***ABCB1***													
		C	T					CC	CT	TT			
rs1045642	Retention *n =* 144	130 (0.59)	92 (0.41)	0.29	0.59	0.03	0.88 [0.56-1.40]	36 (0.32)	58 (0.52)	17 (0.15)	1.23	0.54	0.09
	Dropout *n =* 96	61 (0.56)	49 (0.45)					18 (0.33)	25 (0.46)	12 (0.22)			
		C	T					CC	CT	TT			
rs1128503	Retention *n =* 144	78 (0.37)	132 (0.63)	0.17	0.68	0.03	0.90 [0.54–1.48]	21 (0.20)	36 (0.34)	48 (0.46)	0.54	0.76	0.06
	Dropout *n =* 96	34 (0.35)	64 (0.65)					10 (0.20)	14 (0.29)	25 (0.51)			
		C	T					CC	CT	TT			
rs2032582	Retention *n =* 144	71 (0.33)	147 (0.67)	0.13	0.71	0.02	0.91 [0.55-1.50]	16 (0.15)	39 (0.36)	54 (0.50)	2.31	0.31	0.12
	Dropout *n =* 96	33 (0.31)	75 (0.69)					10 (0.19)	13 (0.24)	31 (0.57)			

### OPRM1/ABCB1 SNP genotypes and negative rate of morphine urine test

As shown in Table 5, the negative rate of morphine urine test was significantly different between the genotypes of rs3192723 and rs562859 (*OPRM1* gene; *p* < 0.05). After adjusting age, sex, and methadone dosage, the significant difference in rs3192723 still existed (*F* = 16.93, *p* < 0.05), but for rs562859, the difference was no longer significant (*F* = 0.43, *p* > 0.05). In addition, the GG genotype (rs3192723) carriers had a significantly lower negative rate of morphine urine test (*p* < 0.05). There was no significant difference between the genotypes of the *ABCB1* gene (*p* > 0.05).

**Table 5:** Negative rate of morphine urine test in different genotypes of the *OPRM1* and *ABCB1* genes.

**Genotypes**	**Negative rate of morphine urine test**			**F**	***p***	**η^2^**
***OPRM1***						
rs3192723	GG (*n =* 83)	G/A (*n =* 71)	AA (*n =* 7)			
	0.69 ± 0.31	0.81 ± 0.27	0.80 ± 0.26	3.55	***0.03***	0.12
rs675026	CC (*n =* 136)	C/T (*n =* 19)	TT (*n =* 2)			
	0.75 ± 0.30	0.73 ± 0.28	0.92 ± 0.00	0.34	0.71	0.04
rs562859	AA (*n =* 93)	A/G (*n =* 59)	GG (*n =* 9)			
	0.80 ± 0.25	0.68 ± 0.34	0.62 ± 0.38	4.25	***0.02***	0.13
rs6912029	GG (*n =* 52)	G/T (*n =* 87)	TT (*n =* 21)			
	0.68 ± 0.32	0.77 ± 0.29	0.81 ± 0.26	1.99	0.14	0.03
rs2236256	CC (*n =* 119)	C/A (*n =* 34)	AA (*n =* 8)			
	0.74 ± 0.31	0.76 ± 0.29	0.82 ± 0.07	0.28	0.76	0.03
rs2236257	GG (*n =* 111)	G/C (*n =* 50)	CC (*n =* 1)			
	0.74 ± 0.31	0.75 ± 0.28	0.91	0.17	0.85	0.03
rs2236258	CC (*n =* 20)	C/T (*n =* 93)	TT (*n =* 48)			
	0.64 ± 0.37	0.76 ± 0.30	0.76 ± 0.26	1.38	0.25	0.08
rs0236259	TT (*n =* 25)	T/C (*n =* 80)	CC (*n =* 55)			
	0.69 ± 0.37	0.75 ± 0.30	0.76 ± 0.26	0.49	0.62	0.05
rs6902403	TT (*n =* 7)	T/C (*n* = 54)	CC (*n* = 97)			
	0.85 ± 0.09	0.77 ± 0.25	0.73 ± 0.32	0.77	0.47	0.06
***ABCB1***						
rs1045642	CC (*n* = 54)	C/T (*n* = 81)	TT (*n* = 26)			
	0.75 ± 0.31	0.76 ± 0.30	0.70 ± 0.30	0.31	0.73	0.04
rs1128503	CC (*n* = 30)	T/C (*n* = 49)	TT (*n* = 71)			
	0.71 ± 0.36	0.74 ± 0.30	0.76 ± 0.29	0.25	0.78	0.03
rs2032582	GG (*n* = 26)	GT (*n* = 49)	TT (*n* = 83)			
	0.76 ± 0.27	0.77 ± 0.31	0.71 ± 0.31	0.59	0.56	0.05

### Factors influencing MMT outcome (compliance and treatment effect)

A logistic regression was used to explore the possible factors that influence MMT compliance and treatment effect. The ASI scores, methadone dosage, drug concentration in plasma, and the SNPs of the *OPRM1*/*ABCB1* genes were included, and a step forward method was used to fit the regression models.

As shown in Table 6, rs562859 (*OPRM1* gene) and free trough concentration were considered in the final model of factors that influence MMT compliance (*p* < 0.05). The dropout risk for participants who carried the AA genotype was 0.58 compared with participants who carried the AG/GG genotype, at the same methadone dosage. If the participants carried the same genotype, the dropout risk decreased to 0.91, when the free trough concentration increased by 1 ng/ml. However, after Bonferroni adjustment, only free trough concentration significantly influenced MMT compliance.

**Table 6:** Logistic regression analysis of factors that influence MMT compliance.

**Variables**	**Regression coefficient β**	**Standard error (S.E.)**	**χ^2^ (Wald)**	**Degree of freedom (df)**	***P***	**Adjusted-p**	**OR**	**95% CI**
rs562859(AA)	−1.770	0.700	6.581	1	0.037	0.074	0.58	0.34~0.97
Free trough concentration	−0.095	0.042	5.175	1	0.023	0.046	0.910	0.839~0.987

As shown in Table 7, the SNPs rs6912029 and rs6902403 of the *OPRM1* gene, age at first use, and the average dosage of methadone were considered in the final model of factors that influence treatment effect (*p* < 0.05). If not, other factors were considered in the model; the rs6912029 GG/GT carriers' negative rate of morphine urine test was 0.066 when compared with the TT carriers, and the rs6902403 CC carriers' negative rate of morphine urine test was 0.095 when compared with the CT/TT carriers. If the participants carried the same genotype and had the same average methadone dosage, the negative rate of morphine urine test increased to 1.118 when the participants' age at first use increased by 1 year. Also, if the participants carried the same genotype and had the same first use age, the negative rate of morphine urine test increased by 1.033 when the average methadone dosage increased by 1 ng/ml. Nevertheless, after the Bonferroni adjustment, the average dosage of methadone was no longer significant in the model.

**Table 7:** Logistic regression analysis of factors that influence negative rate of morphine urine test.

**Variables**	**Regression coefficient β**	**Standard error(S.E.)**	**χ^2^(Wald)**	**Degree of freedom (df)**	***P***	**Adjusted-p**	**OR**	**95% CI**
rs6912029(GG/GT)	−2.714	1.177	5.319	1	0.021	0.042	0.066	0.007~0.665
rs6902403(CC)	−2.355	0.865	7.405	1	0.007	0.014	0.095	0.017-0.517
Age of first use	0.112	0.040	7.788	1	0.005	0.010	1.118	1.034~1.209
Average dosage of methadone	0.032	0.016	4.023	1	0.045	0.084	1.033	1.001-1.066

## Discussion

Methadone is the most widely used medicine for opium dependence. The dose and concentration of methadone in blood is important in MMT. In this study, patients in the retention group had a higher free trough/peak concentration, total trough/peak concentration, and negative morphine urine test. This was consistent with previous studies (Crettol et al., 2005; Ries et al., 2009), indicating that higher methadone drug concentration in plasma was associated with better MMT compliance and a curative effect. However, there was no standard for plasma methadone concentration in China; a previous study conducted in other countries indicated that patients with average plasma methadone concentration of 150–200 ng/ml in 24 h may gain the best curative effect (Ries et al., 2009). In this study, the total trough and peak methadone concentrations of the retention group was 200–350 ng/ml, and they indeed had the better curative effect. In addition, the total trough concentration of dropout group was <200 ng/ml. Low trough concentration may induce withdrawal reaction, which may lead to relapse and dropout from MMT (Ward et al., 1999).

The curative effect of MMT was influenced by many factors, and many previous studies had found an association between the gene and the MMT curative effect; however, conflicting results were obtained (Levran et al., 2013; Isaza et al., 2014). This might be due to the difference in evaluation method and the heterogeneity of the study object. This study used the completion/incompletion of MMT as the compliance index and the negative rate of urine morphine test as the curative effect index, recruited heroin addicts from Shanghai as research objects, and studied the impact of the *ABCB1*/*OPRM1* gene SNPs on the MMT curative effect. The results indicated that the allele and genotype distributions of rs562859 (OPRM1 gene) were different between the retention and dropout groups. However, the difference was no longer significant after adjusting influence factors (age, sex, and methadone dosage), which failed to support the association between the *OPRM1* gene and MMT compliance. Also, the association between rs526859 and negative morphine urine test was not significant after adjusting influence factors. However, individuals who carried the rs3192723 GG genotype had a lower negative rate of morphine urine test. The difference was still significant after adjusting influence factors, which indicated an association between rs3192723 and MMT effect. However, we failed to find an association between the *ABCB1* gene and MMT compliance, which was consistent with previous studies (Crettol et al., 2008; Wang et al., 2012).

As we know, μ opium receptor is the acting site of β-endorphin, morphine, and methadone (Pasternak and Pan, 2013), and it is associated with excitement, analgesia, and withdrawal symptoms of opioids (Pasternak and Pan, 2013). The mechanism of how methadone relieves opium dependence was based on the interaction with opium receptors (Martin et al., 2007). Previous studies showed the association between the *OPRM1* gene and opium addiction (Lötsch et al., 2006; Deb et al., 2010; Kreek et al., 2012) and the association between SNPs of *OPRM1* (mainly rs1799971) and the MMT side effect, methadone dosage, and sensitivity to MMT (Wang et al., 2012; Bauer et al., 2015). The SNP rs526859 located at the second exon of the *OPRM1* gene was a synonymous mutation, which would not result in the sequence change of the protein. Previous studies have proven the relationship between rs562859 and heroin addiction (Smith et al., 2005; Hancock et al., 2015); however, no research focused on the relationship between rs562859 and MMT. Also, our research failed to support this relationship. No previous research focused on rs3192723, whose function was unclear. Our study is the first to find a relationship between rs3192723 and the MMT curative effect; however, further research is still needed to reveal the basis. We failed to find other SNPs of the *OPRM1* gene that had a difference in allele or genotype distribution between retention and dropout individuals, which was consistent with previous studies (Lehotay et al., 2005; Fonseca et al., 2011; Barratt et al., 2012).

The *ABCB1* gene encodes P-glycoprotein, which transfers the substrate out of the cell and plays an important role in the pharmacokinetics of the drug. However, our study failed to find the relationship between SNPs of *ABCB1* and MMT outcomes. Fonseca's study found neither allele nor genotype distribution difference in rs1045642 (*ABCB1* gene) between MMT responders and nonresponders (Fonseca et al., 2011). Crettol's study found that rs1045642 of the *ABCB1* gene slightly influenced methadone metabolism and methadone dose requirement but failed to find an association between rs1045642 or rs2032582 and the methadone curative effect or methadone dosage (Crettol et al., 2008). The studies described above indicated that *ABCB1* might influence drug concentration of methadone in plasma and methadone dosage, other than its direct influence on the methadone curative effect.

The MMT curative effect was influenced by many factors, such as genetic polymorphism, drug dosage, clinical characters of patients, environmental factors, and so on. Previous studies usually focused on the general condition and the external factors, such as age, nation, education level, marital status, occupation, history of drug addiction, methadone dosage, family relationship, friendship with addicts, and publicity, and analyzed the cause of dropout. This study focused on the genetic factors (polymorphism of the *OPRM1* and *ABCB1* genes), methadone dosage, drug concentration in plasma, and ASI score. Also, logistic regression was used to analyze the factors that influence MMT. The results indicated that the SNP rs562859 of the *OPRM1* gene and free trough methadone concentration were the factors that influenced MMT compliance. However, after the Bonferroni correction, only free trough concentration of methadone was negatively correlated with MMT compliance, which means that individuals who had lower free trough concentration of methadone dropped out easily. Trough methadone concentration was the lowest concentration that was needed for the inhibition of withdrawal symptoms. When the trough concentration was too low to inhibit withdrawal symptoms effectively, the patient would feel uncomfortable, turn to drugs, lapse, or relapse, and drop out of MMT. Drug concentration was associated with methadone dosage, which was external and controllable. Relatively, gene was an internal and uncontrollable factor. Our result indicated that rs562859 was associated with MMT outcomes, and that individuals who carried the A allele or AA genotype would have a better compliance rate than those with the G alleles and GG/GA genotypes. However, this association was not significant after the Bonferroni correction. Also, the influence of the rs562859 allele on MMT outcomes was not significant after adjusting influence factors. Therefore, our results failed to support the association between rs562859 and MMT outcomes.

Logistic regression also indicated that the TT genotype of rs6912029 and the CC/CA genotype of rs6902403 (*OPRM1* gene) influenced negative rate of morphine urine test. A previous study found that rs6912029 may be a predictor of treatment outcome in opiate-dependent individuals of Arab descent (Al-Eitan et al., 2012), which was consistent with our results. The SNP rs6912029 is located in the 5′-UTR region, which is known to play crucial roles in post-transcriptional regulation of gene expression. Mutations, which alter the 5′-UTR, may lead to serious pathologies (Barratt et al., 2012). However, further study was needed to explore whether the gene expression was altered in MMT patients. The SNP rs6902403 is located in the intronic region of *OPRM1*. No previous study focused on rs6902403, and our research was the first to find the association between rs6902403 and MMT effect. However, the function of this SNP needed further study. Age at first use and average dosage of methadone also positively correlated with the MMT effect. Nevertheless, the association between the average dosage of methadone and MMT effect was no longer significant after correction. Generally, a higher methadone dosage would result in higher plasma methadone concentration and a better MMT curative effect. There was no previous study that focused on age at first use and negative rate of morphine urine test; our study was the first to find that age at first use was a protective factor of the MMT curative effect. The first use of heroin at a later age and the larger possibility of negative rate of morphine urine test mean that they experienced a better MMT curative effect. If individuals use heroin at an early age, when the brain and body is not fully developed, they would suffer greater damage, which might bring in more serious side effects and worse response to treatment; the effect might extend to adulthood (Fang et al., 2010). In addition, the mechanism was unclear and needed further research.

There were some limitations in this research. Firstly, the follow-up time lasted only for 6 months. Some participants might drop out after this time point, and the results might be different if the follow-up was conducted for a longer time. Secondly, not all participants provided their blood sample. Some participants refused to provide blood, and some participants dropped out before their blood sample was obtained. Hence, there may be some biases because of the missing sample. Thirdly, the standard for tagged SNP selection was minor allele frequency (MAF) and r^2^ based on previous literature, wherefore, the function of some SNPs were not explicit. Fourthly, all the participants were recruited from Shanghai; therefore, the results could only be generalized to Shanghai Han people; a multicenter collaborative study might produce more findings.

## Conclusion

This study found that a higher dosage of methadone/plasma methadone concentration, several SNPs (rs3192723, rs6912029, rs6902403) of the *OPRM1* gene, and the age at first drug use were associated with better MMT outcomes.

## Materials and methods

### Ethics statement

The research protocol was approved by the Ethics Committee of the Shanghai Mental Health Center, and all the subjects signed the informed consent form approved by the Institutional Review Board (IRB) at the Shanghai Mental Health Center (SMHC IRB 2009036).

### Research participants

A total of 240 patients receiving MMT were enrolled from four community clinics (New Pudong, Huangpu, Xuhui, and Hongkou) during April 2010 to June 2011 in this study. All the subjects were aged between 18 and 65 years and had a mean (SD) age of 40.9 ± 8.5 years. In each MMT clinic, two senior psychiatric doctors assessed the subjects. All subjects met the criteria for heroin dependence according to the diagnostic and statistical manual of mental disorders, fourth edition (DSM-IV); they volunteered to take part in this study and did not participate in other studies. The subjects with other psychiatric diagnoses and physical disease, who could not take methadone, were excluded from this study. All subjects completed a self-report form that included basic demographic information (age, gender, years of education, marital status, etc.) and accepted an interview of ASI to collect information on physical health, employment function, use of drugs, use of alcohol, legal problems, family functioning, and mental health. Blood samples were obtained between 4 and 12 weeks after the participants took a stable methadone dosage and were assayed for genotypes. All the samples were obtained from Han Chinese people.

### Clinical outcomes

The data in regard to dosage and time when methadone was taken was obtained from the National Methadone Administration Record and Reporting System. Subjects were divided into two groups (retention and dropout) at the end, in terms of whether or not they completed the 6-month MMT. If the subject failed to take methadone for 7 days continuously, he/she would be assigned to the dropout group (Xiaoli et al., 2014). Urine samples were obtained every 2 weeks for morphine urine test. If the participant was absent for or refused to take the test, the result of the urine test was recorded as positive. The negative rate of morphine urine test was calculated for every subject in the end (Fang et al., 2010). The standards for the division of retention/dropout group and the calculation of negative rate of morphine urine test were in reference to previous studies and were modified according to reality.

### Selection of SNPs and genotyping of DRD1 polymorphism

**Figure 1:**
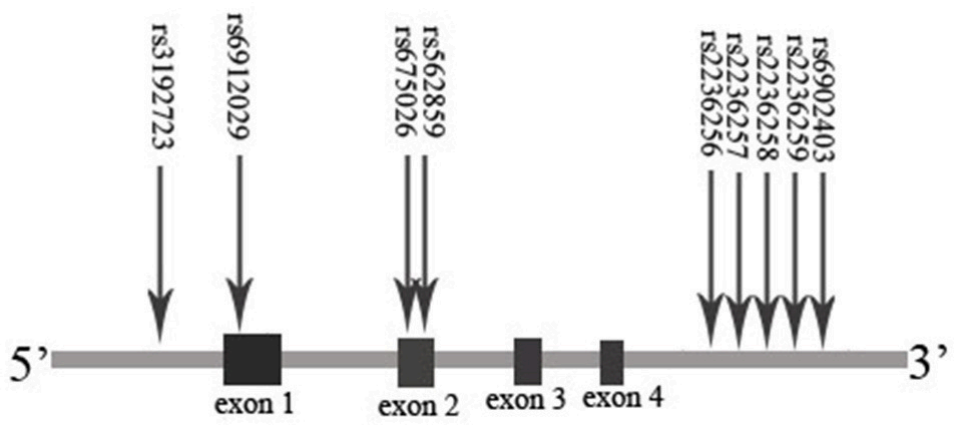
Sites of selected SNPs on the *OPRM1* gene.

**Figure 2:**
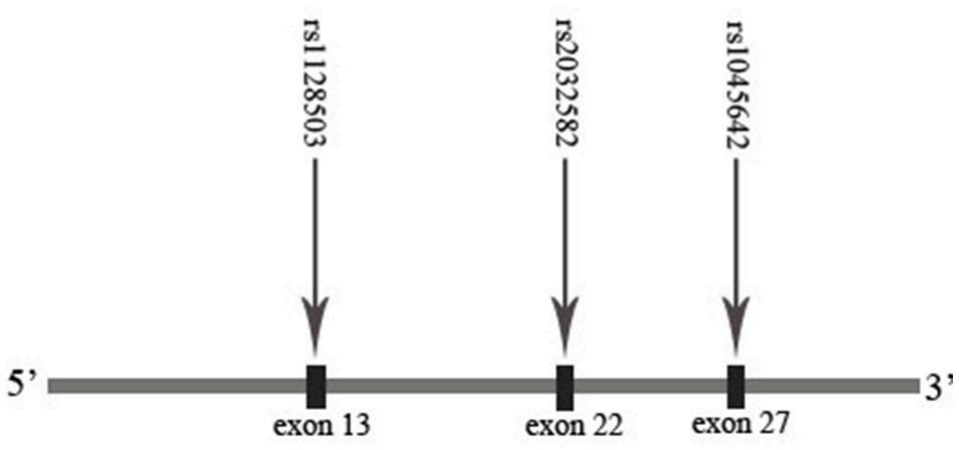
Sites of selected SNPs on the *ABCB1* gene.

The extraction of DNA was performed using a modified phenol/chloroform method (Peng et al., 2013). Tagged SNPs for *OPRM1* and *ABCB1* were selected from the separated regions of Chr6: 154331631…154568001 and Chr7: 87133179…87342639 in the HapMap database for the Han Chinese people. Tagged SNP was defined as having an MAF > 0.10 and *r*^2^ > 0.8. Nine tagged SNPs of *OPRM1* (rs3192723, rs675026, rs562859, rs6912029, rs2236256, rs2236257, rs2236258, rs2236259, and rs6902403) and three SNPs of *ABCB1* (rs1045642, rs1128503, and rs2032582) were selected (Figures 1, 2). For genetic polymorphism genotyping of *OPRM1* and *ABCB1*, SnapShot Genotyping Assay (Applied Biosystems, Foster City, California) was performed on the ABI Prism 3130X1 sequence detection system. In order to calculate genotyping error, 5% random DNA samples were genotyped twice for each SNP. The genotyping accuracy was 100%.

### Statistical analyses

Power and sample size calculations were computed with the G^*^ Power software (version 3.1.3, Franz Faul, Germany). The minimum sample size was estimated using the frequencies observed in the retention and dropout participants with α = 5% and β = 0.1. An online software program (SHEsis; http://analysis.bio-x.cn/myAnalysis.php; Shi and He, 2005) was used to test Hardy–Weinberg equilibrium and linkage disequilibrium (LD). Analysis of variance (ANOVA) was used to estimate the difference between groups of indexes described as follows: clinic features of participants; ASI; methadone dosage, drug concentration; negative rate of morphine urine test in retention and dropout participants; negative rate of morphine urine test in different genotypes. Least significant difference (LSD) was used for the *post hoc* test. Cohen's d was used to evaluate the effect size. Odds ratios (ORs) were used to measure the association between the *OPRM1*/*ABCB1* alleles and methadone treatment compliance. Φ and η^2^ was used to evaluate the effect size. Unconditional logistic regression models were used to obtain maximum likelihood estimates of the ORs and their 95% confidence intervals (CIs). Covariance analysis was used to control the possible influence factors. Unconditional logistic regression analysis (step forward) was used to analyze the factors that influence the compliance of MMT and negative rate of morphine urine test. Bonferroni correction was used for multiple comparisons. Statistical analyses were performed using SPSS 23.0 (SPSS Inc., Chicago, IL, USA). All tests were two-tailed, and the significance level was set at 0.05.

## Author contributions

SP was responsible for statistical data analysis and manuscript writing. HJ and JD were responsible for the experimental design. SL and SP were responsible for conducting the experiments. SY was responsible for the experiment guide. MZ was responsible for the overall control.

### Conflict of interest statement

The authors declare that the research was conducted in the absence of any commercial or financial relationships that could be construed as a potential conflict of interest.

## Abbreviations

OPRM1: μ-opioid receptor gene
ABCB1: multidrug resistance 1 gene
MMT: methadone maintenance treatment
SNPs: single nucleotide polymorphisms
ASI: addiction severity index
IRB: Institutional Review Board
DSM-IV: the diagnostic and statistical manual of mental disorders, fourth edition
LD: linkage disequilibrium
ANOVA: analysis of variance
OR: odds ratios
CI: confidence intervals.
